# Investigating memory for faces based on emotional contextual information

**DOI:** 10.3758/s13421-025-01830-w

**Published:** 2026-03-30

**Authors:** Brandon H. Edwards, Delaney Walden, Paul A. Bloom, Katherine R. Mickley Steinmetz

**Affiliations:** 1 Department of Psychology, University of North Carolina at Greensboro, Greensboro, NC USA; 2 Department of Psychology, Wofford College, Spartanburg, SC USA; 3 Department of Psychology, Medical University of South Carolina, Charleston, SC USA; 4 Department of Psychology, Columbia University, New York, NY USA

**Keywords:** Memory, Context, Emotion, Faces

## Abstract

Contextual affective information can influence recognition memory. However, the influence of the order of this information on memory has rarely been studied. To investigate this, a series of faces were shown along with contextual person-based information. Each face was shown twice, manipulating the order of the affective contextual information: (1) emotional then neutral, (2) neutral then emotional, or (3) neutral and then neutral. The first study included negative contextual information and the second included positive information. One week later a recognition test was given for the faces. In both the negative study and the positive study, recognition memory was uninfluenced by condition, suggesting that the order in which affective information is presented is largely irrelevant. Further, exploratory Bayesian analyses revealed that faces paired with the highest arousal sentences at encoding were the least likely to be recognized at retrieval, which may represent a biasing of attention towards the high-arousal sentence and away from the low-arousal face.

**Supplementary Information:**

The online version contains supplementary material available at 10.3758/s13421-025-01830-w.

## Introduction

The ability to remember people and person-information is important to facilitate social interactions. However, an unanswered question is how the order in which one learns new information influences the memories of people that we encounter. One may immediately learn affective information, or one may first learn neutral information and then learn affective information about someone. This is relevant to many real-life experiences in which order of information acquisition may matter. For example, would you remember someone better if you first encountered them robbing a bank, or if you had first seen them at the grocery store the week before picking out oranges? In a legal context, having these kinds of data would be especially important in suspect identification and other forms of eyewitness testimony. In addition, this is relevant to how first impressions may be related to memory in subsequent social encounters.

### Affective contextual information influences memory

In general, affective information is better remembered than neutral information (see LaBar & Cabaza, 2006). However, when one learns affective information about someone, the affective information is contextual information, bound to the memory for that individual. Past studies have shown that affective contextual information at encoding can influence later memory for neutral information (Maratos & Rugg, 2001; Smith et al., 2004). Evidence of this ability to link affective context to neutral information comes from studies that have found that neutral words or objects were associated with enhanced brain activity when they had been encoded in negative contexts as opposed to neutral contexts, perhaps corresponding to increased post-retrieval processing.

There is also evidence that affective person-based information paired with faces at encoding can influence implicit and explicit memory. For example, neutral faces previously paired with negative person-based information at encoding were later rated as more negative when presented alone (Falvello et al., 2015). In addition, Mattarozzi and colleagues (2019) found that person-information at encoding increased memory for faces. Participants were presented with faces either alone or faces paired with behavioral descriptions that were positive, negative, or neutral. A recognition memory test was given either immediately or after a 1-week delay. Despite a lower perceptual load and a longer viewing time for the faces alone, participants were more likely to remember the faces at both time-points if they had previously been paired with behavioral information. In addition, after a 1-week delay, memory was specifically enhanced for faces which had been paired for positive and negative information as compared to those paired with neutral information. This suggests that person-related contextual information may be integrated into memory for the faces and may lead to a more complex memory (Todorov & Uleman, 2004). From a functional perspective, this may be especially beneficial in guiding future social interactions.

Thus, affective person-information at encoding can influence memory. However, an open question is how the order in which someone learns affective information influences memory. In order to examine this in the current study, participants were shown a series of faces. Each face was presented twice, (1) first paired with an emotional sentence, then a neutral sentence (Emotional-Neutral), (2) first paired with a neutral sentence, and then with an emotional sentence (Neutral–Emotional), or (3) first paired with a neutral sentence, and then another neutral sentence (Neutral–Neutral). In Experiment 1, the emotional sentences were negative, and in Experiment 2 the emotional sentences were positive. In this way, the current study builds upon Mattarozzi and colleagues’ (2019) by expanding their experimental design to consider how the order of presentation may influence memory.

### Order influences social judgments

Past studies have shown that the order in which someone learns person-based information influences their social judgments. For example, studies have shown that implicit first impressions may be especially difficult to undo, even in the face of updated information. (Boucher & Rydell, 2012). This is especially the case when the initial impression is negative rather than positive (see Baumeister et al., 2001; Rozin & Royzman, 2001). The Associative–Propositional Evaluation Model suggests that this may occur because of the formation of associative networks when initial information is learned, pairing the memory with related contextual and semantic information (Gawronski & Bodenhausen, 2011). Once formed, new information is more easily learned if it fits within these networks. In the current study, this may suggest that when a negative piece of information is learned first, the lingering implicit impressions may lead to increased memory for the face. However, these studies have not examined the influence of affective information which comes second, or how these impressions influence memory.

### Order of affective stimuli influences memory

Though the influence of order on affective person-based contextual information has not been studied, there is evidence that the order of affective information itself may influence memory even when there is a delay between encoding episodes. For example, Tambini, Rimmele, and colleagues (2017) used a paradigm where a block of emotional stimuli was either followed by a 6-min rest period and then a block of neutral stimuli, or vice versa. A recognition test was administered 6 h later, and it was found that neutral stimuli that followed the block of emotional stimuli (Emotional–Neutral) were more likely to be remembered than neutral stimuli that either preceded emotional stimuli (Neutral–Emotional) or succeeded another block of neutral stimuli (Neutral–Neutral). This emotional carryover effect when emotional stimuli were presented before neutral stimuli (Emotional–Neutral) may have been induced by the lingering arousal response evoked by the emotional stimuli, as evidenced by the increased skin conductance response and reinstatement of emotion-related brain activity for the neutral stimuli that followed emotional stimuli. This may relate to the Emotional–Neutral condition in the current study, indicating that the emotional information from the first face presentation may carry over to the second face presentation.

In addition to these anterograde effects on memory when the affective information comes first, order may influence memory for neutral stimuli that come before emotional stimuli, which is relevant to the Neutral–Emotional condition in the current study. Knight and Mather (2009) found that memory for preceding neutral information is enhanced by an emotional stimulus if it is linked to or predicts the following stimulus, and if memory was tested with a week between encoding and retrieval as opposed to in the same testing session. In the current study the face remains constant across presentations, thus linking the two stimulus presentations. Therefore, due to the repeated face presentation with varying sentences, it may be more likely to boost rather than interrupt consolidation processes, leading to a more stabilized memory trace (Takashima et al., 2007). The Neutral–Emotional condition may benefit from increased memory for the face if the face is still being consolidated and the following emotional sentence increases the consolidation of that face.

Together, these results indicate that over the course of a temporal stream of information, emotional stimuli can modulate both encoding and post-encoding processes in order to influence what is remembered.

### The influence of valence on binding memory to context

Another factor that likely influences the effect of order on memory is the valence (positive or negative) of the contextual information. As mentioned previously, negative first impressions are more difficult to undo (see Baumeister et al., 2001; Rozin & Royzman, 2001). In addition, studies have shown differences in processing positive versus negative stimuli even when matched on arousal (e.g., Delplanque et al., 2004). Specifically, positive moods or positive stimuli are associated with a widened scope of attention (see Conway et al., 2013) while negative moods or negative stimuli are associated with more narrowed attention (Easterbrook, 1959). These differences in processing at encoding have been linked to changes in how stimuli of different valences are remembered. Negative stimuli are associated with a greater sense of recollection and are encoded with additional visual processing areas of the brain, whereas positive stimuli are more likely to be associated with a general sense of familiarity, episodic and semantic retrieval, and self-referential processing (Mickley & Kensinger, 2008; Ochsner, 2000). According to the *NEVER Forget Theory*, negative information is also associated with enhanced recapitulation of sensory details over time (Bowen et al., 2018) yielding a vivid, but narrow, memory. These findings indicate that there are concrete differences in the processing of and memory for positive as compared to negative information.

However, will these differences remain when person-based affective *contextual* information is paired with faces? In essence this is a question of whether the affective information will be bound to the neutral face using associative memory processes. In word-pair studies, negative words have been shown to impair associative memory, meaning that the neutral word is less likely to be remembered if it had been paired with a negative word (Madan et al., 2012, 2017). On the other hand, positive information is associated with enhancements in associative memory, but only if both words were positive at encoding (Madan et al., 2019; Zimmerman & Kelley, 2010). Some similar effects have also been shown in recognition memory tests for memory of scenes, where for positive scenes participants were able to remember both central and peripheral details, while negative scenes were limited to memory for central details (Chipchase & Chapman, 2013; Smith et al., 2004; Yegiyan & Yonelinas, 2011). However, other studies have found that people are more likely to remember emotional aspects of scenes and are more likely to forget backgrounds for scenes that include positive as well as negative stimuli (Mickley Steinmetz et al., 2012; Mickley Steinmetz & Kensinger, 2013). Though these results are mixed, they may indicate that it is easier to bind positive information to neutral information than to bind negative information to neutral information. Because of this, it is possible that broadened attention for positive information may lead to a stronger link between face and person-information and be more influenced by temporal order of the contextual information as compared to negative information.

### The influence of arousal on binding memory to context

Arousal of the contextual information may also influence memory binding. The *arousal biased competition* (ABC) model suggests that when there are competing memory representations such as trying to remember a face and contextual person-based information that is paired with it, arousal modulates the strength of the memory representation based on top-down goals or bottom-up stimulus features (Mather & Sutherland, 2011). High-priority memory representations lead to more attention and stronger memories. For example, this priority-driven selective enhancement was shown by Sakaki and colleagues (2014), who found that memory for an emotionally arousing image led to impaired memory for a preceding neutral item that was low priority, but facilitated it when they prioritized that item. The neural mechanisms have been explored and are explained in the *Glutamate Amplifies Noradrenergic Effects* (GANE) theory, which suggests that norepinephrine (NE) hotspots lead to greater metabolic resources channeled to memory representations of high-priority representations, while low-priority representations are inhibited (Mather et al, 2016; Turkileri & Sakaki, 2017). High glutamate concentrations are associated with cortical regions that are strongly activated. Arousal leads to a release of NE from the locus coeruleus, which acts to up-regulate activity in areas where glutamate is high (NE hotspots) and down-regulate activity where glutamate is low via inhibitory adrenoreceptors. In this way, high-arousal person-based information may lead to enhanced memory for a face only if it is goal relevant.

### The current study

The current study presented faces paired with affective and neutral person-based information and manipulated the order of person-based information for each face as follows:

1. first paired with an emotional sentence, then a neutral sentence (Emotional–Neutral),
2. first paired with a neutral sentence, and then with an emotional sentence (Neutral–Emotional), or
3. first paired with a neutral sentence, and then another neutral sentence (Neutral–Neutral).

The first, preregistered, experiment utilized negative affective information, while the second, exploratory, experiment utilized positive affective information.

For the Negative experiment, we hypothesized that recognition memory would be best for faces that were first paired with negative and then paired with neutral sentences (Negative–Neutral), second best for faces that were first paired with neutral and then paired with negative sentences (Neutral–Negative), and worst for faces that were paired with neutral sentences each time (Neutral–Neutral). We hypothesized that faces in the Negative–Neutral condition may be remembered best because the second presentation of the face may evoke enhanced processing, as it was linked to the emotional content from the previous presentation (Tambini, Rimmele, et al., 2017). Faces paired in the Neutral–Negative condition were hypothesized to be remembered better than those in the Neutral–Neutral condition due to a potential boost in consolidation and attention when a face that was previously paired with neutral information is paired with negative information (Knight & Mather, 2009; Takashima et al., 2007), but remembered worse than faces in the Negative–Neutral condition due to the latter benefiting from an additional dose of enhanced affective processing.

For the Positive experiment, we hypothesized that memory for the face would be enhanced when the emotional information comes second, following neutral information (Neutral–Positive), as compared to both other conditions. This may be because of a boost in the consolidation of the previously presented face (Knight & Mather, 2009) or the enhanced processing due to the unexpected news that this person, previously assumed to be neutral, possesses positive qualities. This enhanced processing may be especially likely for positive information due to the increased perceived reward value for unexpected rewards (Hollerman & Shultz, 1998).

Lastly, an exploratory research question targeted how the initial experience of valence and arousal influenced memory for each face. To address this question, we employed Bayesian multilevel logistic regression to pair the continuous arousal rating of each individual trial with whether it was later remembered or forgotten. This allows individual participant ratings to guide the analyses instead of relying on averaged pre-categorized data.

#### **Data availability**

Materials, data, and analysis code may be found here: https://osf.io/rhac3/.

## Experiment 1: The negative experiment

### Method

#### Participants

Target sample size was determined a priori using G*Power (Faul et al., 2007). The effect size was taken from Mattarozzi and colleagues (2015), which demonstrated a η_p_^2^ of.37 for an interaction between emotional context (neutral, pleasant, unpleasant) and type of memory (face familiarity, face and valence of information, face and content of information), such that while participants were more likely to recall the valence of and content of the information paired with a face in emotional contexts, they were more likely to only recognize the faces as familiar in neutral contexts. This effect size was chosen because of its relation to our hypothesis that recognition memory for a face would be greater when paired with a negative contextual sentence than when paired with two neutral sentences alone. Using an alpha of.05 and a power of.95, G*Power indicated with an actual power of.952 that 26 participants would be needed to detect a similar effect with an effect size of this magnitude. Further, a simulation-based power analysis indicated that our study, with *N* = 90 new trials and *N* = 90 old trials per participant, would be 80% powered to detect an η_p_^2^ of approximately.175, substantially smaller than the value of.37 observed by Mattarozzi and colleagues (2015) (see Electronic Supplementary Materials (ESM)). Participants included 32 undergraduate students (16 female), ranging from 18 to 25 years old (*M* = 20.688, *SD* = 0.998). Participants had normal or corrected-to-normal vision and did not have psychiatric or learning disorders. This study was approved by the Wofford College Institutional Review Board.

#### Materials

Face stimuli included 180 forward-facing pictures of Caucasian individuals (90 male) selected from the FERET Color Database (Phillips et al., 1998). Ninety faces were used at encoding and 90 new faces were included as lures at retrieval. The faces used at encoding and retrieval were counterbalanced across participants. The faces in these sets were selected to contain an equal number of young and old individuals, all wearing ordinary clothing. For both male and female faces, the same proportion of individuals wore glasses (one-ninth of the set). These features, in addition to the presence of beards in males, were matched between encoding and lure items.

Negative and neutral sentences were created using words from the Affective Norms of English Words (ANEW) database (Bradley & Lang, 1994). First, words were chosen such that negative words had higher ratings of valence (*t*(178) = 17.978, *p* <.001) and arousal (*t*(178) = 14.245, *p* <.001) than neutral words, but did not differ by word frequency from neutral words, *t* < 0.4, *p* >.7. Once words were selected, a sentence was then created using each word. The majority of sentences consisted of a subject, indicated by a pronoun (i.e., “he” or “she”), performing an action, with the chosen ANEW word acting as a verb. The remainder of the sentences used an ANEW word to describe a subject’s character or used an ANEW word as a location. Two versions of each sentence were created: one with male pronouns and one with female pronouns. A list of all sentences used is provided in the Appendix.

A pilot study was conducted to assess the emotional valence and emotional arousal of each sentence. Participants included 20 undergraduate students. Each participant rated 170 sentences. The use of male or female pronouns in the sentences was counterbalanced across participants. Participants ranked these sentences on valence and arousal using self-assessment manikins (Bradley & Lang 1994): for valence, 1 = negative/unpleasant and 9 = positive/pleasant, and for arousal, 1 = calming/soothing and 9 = exciting/agitating.

Using these pilot ratings, 60 negative descriptions were chosen, and 120 neutral descriptions were chosen such that negative sentences were rated as significantly more negative (*M* = 1.797, *SE* = 0.069) than neutral sentences (*M* = 5.244, *SE* = 0.034), *t*(178) = 50.329, *p* <.001). In addition, negative sentences were rated as significantly more arousing (*M* = 7.409, *SE* = 0.107) than neutral sentences (*M* = 3.214, *SE* = 0.035), *t*(178) = 46.375, *p* <.001). Negative and neutral sentences were also selected such that they did not differ in sentence length or word frequency of the ANEW word, *t* < 0.4, *p* >.7.

Other materials included the 19-item version of the Beck Depression Inventory (BDI-I; Beck et al., 1961) and the 21-item Beck Anxiety Inventory (BAI; (Beck et al., 1988). E-Prime (version 3.0, Psychology Software Tools, Inc., Pittsburgh, PA, USA) was used to present faces and sentences at both encoding and retrieval.

#### Procedure

Prior to arrival, all participants completed electronic demographic surveys and digital versions of the Beck Depression Inventory (Beck et al., 1961) and the Beck Anxiety Inventory (Beck et al., 1988).

**Fig. 1:**
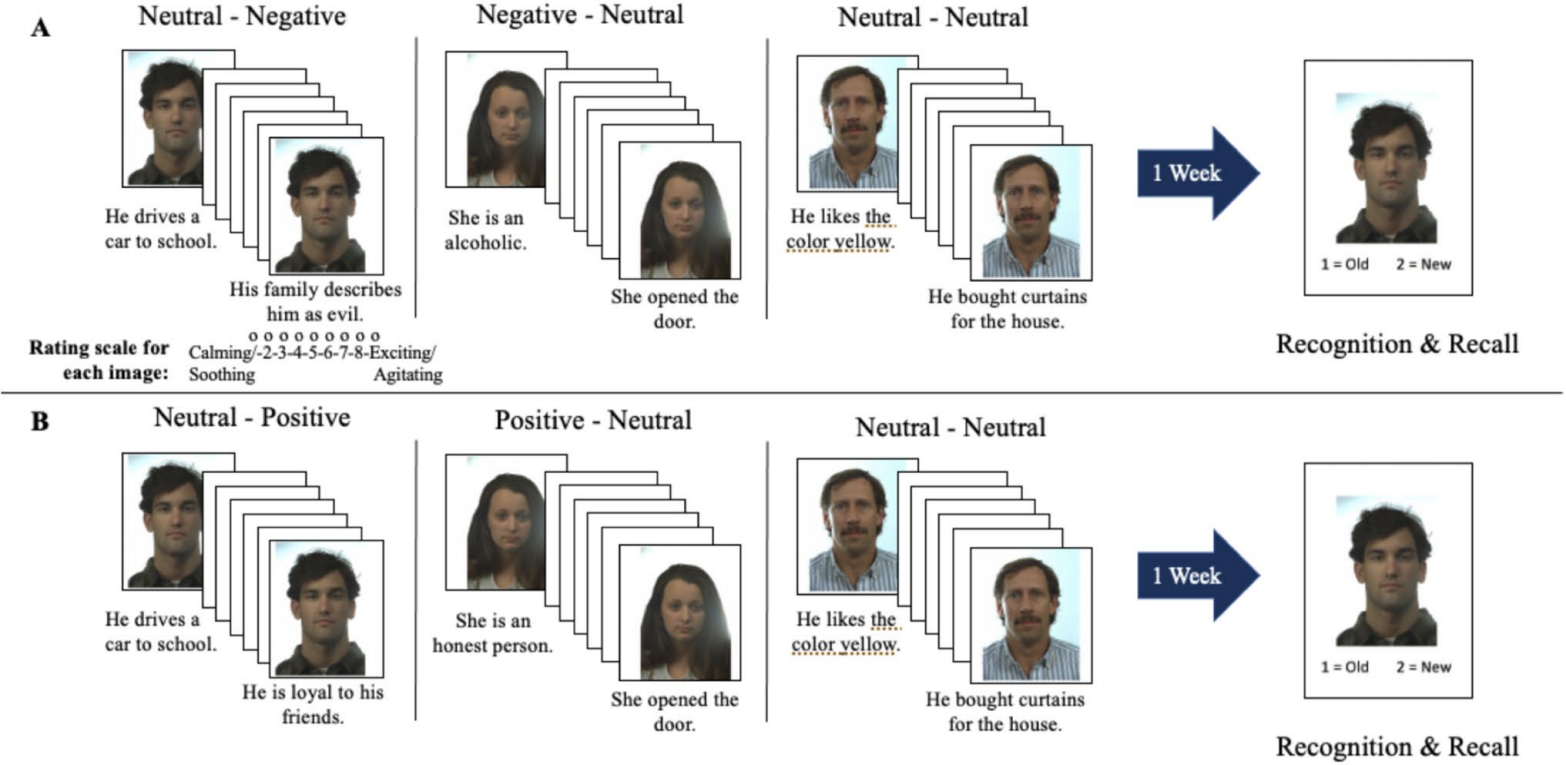
Study design for the negative study (**A**) and positive study (**B**). During encoding, 90 faces were presented twice each with five faces in between presentations. Each face was paired with a verbal descriptor of positive, negative, or neutral depending on the condition. Participants were asked to look carefully at each face and read each sentence and make an arousal rating from calming/soothing to exciting/agitating for each face/sentence pair. Participants were asked to rate the combination of the face and the sentence. Examples of each of the three conditions are presented here. One week later, at retrieval 180 faces were presented with no verbal descriptors; 90 faces were from encoding and 90 new faces were used. If participants recognized a face as old, they were prompted to freely recall the sentences associated with the face

Once in the lab, participants began with an encoding session in which they were presented with a series of individual faces with neutral expressions paired with a single verbal sentence descriptor below each face. Participants were asked to rate each sentence on arousal on a scale from 1 (calm/soothing) to 9 (exciting/agitating). Specifically, participants were told, “Please look at each face and sentence carefully and try to remember both, as you will be tested on your memory for each when you come in next week.” Participants were asked to rate the combination of the face and the sentence. The timing and design were based on Mattarozzi and colleagues (2019). Each face/sentence pair remained on the screen for 8 s, followed by the display of a fixation cross for 1 s. Each face was presented twice, each time with a different verbal descriptor. The second appearance of a specific face occurred six trials later, such that five faces appeared in-between the two presentations. Alternatively, this procedure can be thought of as selecting six random faces, showing each face twice, in the same order, for a total of 12 trials, then selecting six new faces and repeating. There were 30 faces for each condition, yielding a total of 90 faces at encoding. Since each face was presented twice, there were 180 face presentations at encoding. Each face was randomly assigned to a condition for each participant, and sentences were further randomly matched with each face for each participant, such that a random face was presented with a random sentence for each participant. There were three different conditions:

1. a face with a negative descriptor the first time and a neutral descriptor the second time (Negative–Neutral),
2. a face with a neutral descriptor the first time and a negative descriptor the second time (Neutral–Negative),
3. a face with a neutral descriptor the first time and a neutral descriptor the second time (Neutral–Neutral) (see Fig. 1).

Although the gender of each face was randomly chosen, it was kept equal across conditions, such that 15 were always female and 15 were always male in each condition.

One week later, participants engaged in a recognition memory task and free-recall test. For the recognition memory task, the 90 faces from the first session were randomly intermixed with 90 new faces (lures) and participants were asked to make an old/new judgment. For each face that the participant judged as old, participants wrote down as much as they remembered from the descriptors paired with the face at encoding, immediately after making each old/new judgment. Lure and study images were counterbalanced across participants such that the 90 faces used at encoding were instead used as lures for half of the participants.

#### Data analysis

Recognition memory was scored by determining a hit percentage and a false alarm percentage. Hit percentage was calculated by dividing the number of correct recognitions (old faces identified as old) by the total number of faces in each category. False alarm percentage was calculated by dividing the number of new faces identified as old by the total number of new faces. Since false alarms by definition consisted of pictures that were only shown at retrieval (faces that participants falsely recognized as old), they did not have an encoding condition. Thus, hits are used as the dependent variable in all analyses. Corrected recognition percentage, calculated by subtracting the false alarm rate from the hit percentage, yielded all of the same results as using hits as the dependent variable. See Table 1 for hits and false alarms presented separately.

**Table 1:** Recognition memory for each experiment divided by hits and false alarms

	Experiment 1: Negative Study		Experiment 2: Positive Study	
	*M*	*SE*	*M*	*SE*
Emotional–Neutral Hits	0.579	0.031	0.584	0.031
Neutral–Emotional Hits	0.577	0.036	0.605	0.028
Neutral–Neutral Hits	0.551	0.034	0.57	0.035
False Alarms	0.049	0.012	0.115	0.023

Hits separated by category were used in all ANOVAs. When false alarms were subtracted from hits, all results remained the same

*M =* mean, *SE* = standard error of the mean

Recall memory was scored by giving 1 point for correct recall of the sentence and.5 for partially correct recall. The total number of correct recalls was multiplied by one. The total number of partially correct recall was multiplied by.5. The sum of these two scores was added together and then divided by the total possible number of sentences shown. Recall memory was at floor, and as such the data are not reported in this article.

For all analyses of variance, depression and anxiety scores were used as a covariate. This is because studies have shown that depression and anxiety can influence emotional memory (Coles & Heimberg, 2002; LeMoult & Gotlib, 2019).

#### Bayesian modeling of arousal

Using the rstanarm package in the R computing environment (Goodrich et al., 2023), a Bayesian multilevel logistic regression model was applied to determine the association between the arousal (Experiment 1) or valence (Experiment 2) rating for each sentence and recognition memory on a trial-by-trial basis. This allowed us to examine if the arousal or valence rating for either the first or the second sentence in a particular face/sentence pair predicted recognition memory for that face. The advantage of this analysis is that each participant’s experience of each stimulus can be considered, as opposed to averaging memory across a particular category (Bloom et al., 2022; Gelman, 2006). The outcome variable was recognition memory of faces previously seen (1 = remembered, 0 = forgot). Rating scores were mean-centered within-participant, such that each participant had a mean of 0 for each predictor variable, to facilitate interpretation of the interaction parameters. For all parameters of interest (all beta coefficients), package default weakly informative priors were used, namely normal distributions with standard deviations of 2.5 on a standardized scale. For all models, we fit four chains of 2,000 sampling iterations (1,000 warmup) each for a total of 4,000 post-warmup samples. Models achieved adequate convergence, as effective sample sizes were > 1,000 for all parameters of interest, and Rhat statistics for all fixed effects were below 1.01. While Bayesian analyses, strictly speaking, do not speak to “significance” in a frequentist sense, whether 95% posterior intervals excluded 0 was used as a rough proxy for significance (i.e., intervals not overlapping 0 were considered significant). Extraction and transformation of posterior draws after models were fit was done using the tidybayes package and the tidyverse collection of packages in R (Kay, 2023; Wickham et al., 2019). All result figures were created using ggplot2. Odds ratios were calculated with respect to a 1-point increase in arousal relative to each participant’s mean.

### Results

#### Validity check of arousal ratings

As a validity check, a paired-samples *t*-test was conducted on each participant’s arousal ratings during the experiment, comparing ratings of negative sentences paired with neutral faces to neutral sentences paired with neutral faces. As intended, participants rated negative sentences as higher in arousal (*M* = 7.156, *SE* = 0.223) than neutral sentences (*M* = 3.141, *SE* = 0.183), *t*(31) = 16.875, *p* <.001, *d* = 2.983.

#### Effect of order of sentence presentation

In order to examine the influence of sentence order on recognition memory, a one-way Condition (Negative–Neutral, Neutral–Negative, Neutral–Neutral) repeated-measures ANOVA was conducted on recognition memory (hits). This analysis revealed no significant main effect of Condition, *F*(2,62) = 0.835, *p* =.439, η_p_^2^ =.026.

To examine whether accounting for anxiety or depression symptomology affected the influence of sentence order on memory, two one-way Condition (Negative–Neutral, Neutral–Negative, Neutral–Neutral) repeated-measures ANCOVAs were conducted on recognition memory (hits), one using anxiety (BAI) scores as a covariate and the other using depression (BDI) scores as a covariate. The first ANCOVA, using BAI scores as a covariate, revealed no significant main effect of Condition, *F*(2, 60) = 0.248, *p* =.781, η_p_^2^ =.008. Likewise, no significant interaction between Condition and BAI scores was found, *F*(2, 60) = 1.713, *p* =.189, η_p_^2^ =.054. The second ANCOVA, using BDI scores as a covariate, found no significant main effect of Condition, *F*(2, 60) = 0.129, *p* =.879, η_p_^2^ =.004, and no significant interaction between Condition and BDI scores, *F*(2, 60) = 1.535, *p* =.224, η_p_^2^ =.049.

### Exploratory Bayesian analysis of arousal and memory

**Fig. 2:**
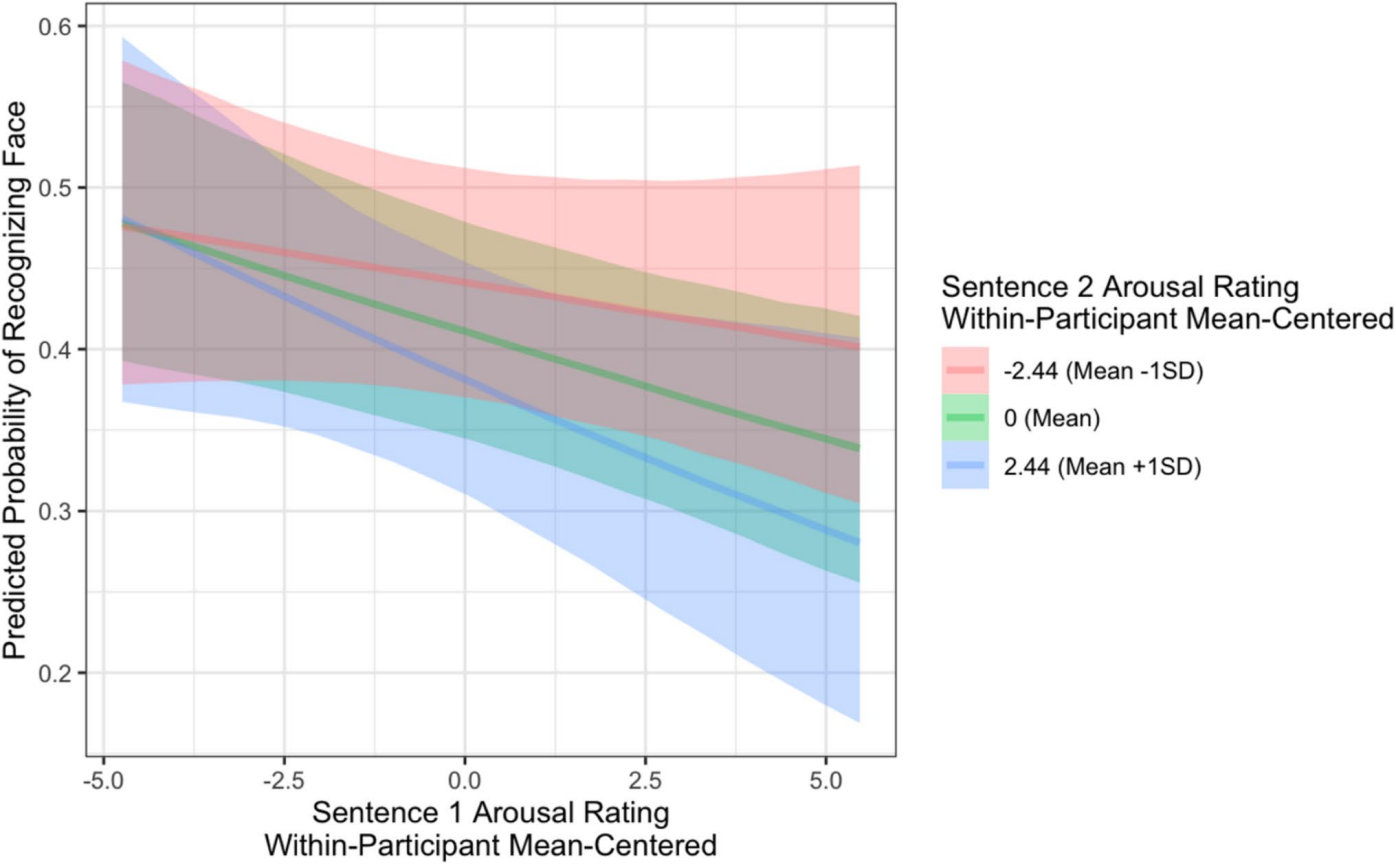
Predicted probability of recognizing a face based on arousal ratings for sentences 1 and 2. Predicted probability of a participant recognizing a given face in the negative study, based on their arousal ratings for sentences 1 and 2 paired with the face. Bayesian analyses suggest that faces paired with the most arousing sentences were the least likely to be recognized. The y-axis represents the probability of successfully recognizing a face as “old.” The x-axis represents the individuals’ arousal rating of the first sentence of a sentence pair. The colored lines represent the arousal of the second sentence of a sentence pair, with pink indicating low arousal, green indicating moderate arousal, and blue indicating high arousal ratings

Arousal of the sentence presented at first encoding was negatively associated with the probability of recognition (β = −0.057, 95% PI [−0.104, −0.016]; OR = 0.95, 95% PI [0.92, 0.98], see Fig. 2). Arousal of the sentence presented at second encoding was also associated with the probability of recognition (β = −0.051, 95% PI [−0.098, −0.009], OR = 0.96, 95% PI [0.93, 0.99]). However, there was no interaction between arousal of sentences 1 and 2 and the probability of recognition (β = −0.011, 95% PI [−0.035, 0.012]).

## Experiment 2: The positive experiment

Of the studies that have examined the temporal order of stimuli, most have focused on negative stimuli. However, studies have shown that positive stimuli can lead to broadened attention (e.g. Conway, et al., 2013), familiarity as opposed to recollection (e.g. Ochsner, 2000), and better associative memory (e.g. Madan et al., 2012). For these reasons, the second experiment explored the influence of temporal order of contextual affective information using the same paradigm as in the first experiment only with positive contextual stimuli instead of negative contextual stimuli.

### Method

#### Participants

Sample size calculations and exclusion/inclusion criteria were the same as in the Negative experiment. Participants included 32 undergraduate students (16 female), ranging in age from 18 to 22 years (*M* = 19.78, *SD* = 1.408).

#### Materials

The same 180 face stimuli included in the Negative experiment were also used in the Positive experiment. Positive and neutral sentences were selected in a similar way to that in Experiment 1: positive ANEW words were first chosen, while the same neutral words were used from Experiment 1. The selected positive ANEW words were significantly more positive (*t*(177) = 18.281, *p* <.001) and higher in arousal (*t*(178) = 2.011, *p* =.046) than the neutral words, without differing in word frequency, *t*(178) *=*.993, *p* =.332. Then, positive sentences were created, and valence and arousal ratings were collected via a pilot study with 20 undergraduate students who rated 170 positive and neutral sentences. Using these ratings, 60 positive sentences were selected by evaluating their reported valence and arousal in comparison to the previously selected neutral sentences. Positive sentences were selected to be more positive (*M* = 7.704, *SE* = 0.778) than neutral sentences (*M* = 5.679, *SE =* 0.068),* t*(178) = 35.009, *p* <.001. In addition, positive sentences were also rated higher in arousal (*M* = 4.662, *SE* = 0.250) than neutral sentences (*M* = 4.247, *SE* = 0.076) (*t*(178) = 22.107, *p* <.001) without differing in sentence length, *t*(178) = 1.579, *p* =.116.

#### Procedure

The procedure was largely the same as in the Negative experiment except that participants were asked to rate the valence of each sentence on a scale from 1 (unpleasant) to 9 (pleasant). Valence ratings were selected instead of arousal because of prior research suggesting that while negative words impair associative memory (Madan et al., 2012, 2017), positive words enhance associative memory only when both words are perceived as positive at encoding (Madan et al., 2019; Zimmerman & Kelley, 2010). Furthermore, our hypotheses for positive stimuli were based on the intensity of positive valence (see Conway et al., 2013). There were three different conditions: (1) a face with a positive descriptor the first time and a neutral descriptor the second time (Positive-Neutral), (2) a face with a neutral descriptor and then a positive descriptor (Neutral–Positive), (3) a face with a neutral descriptor and then a neutral descriptor (Neutral–Neutral).

#### Data analysis

Recognition memory was computed in the same way as the prior experiment. Again, recall memory was at floor, and as such the data are not reported in this article. Bayesian analysis was conducted in the same manner as in Experiment 1, with the exception being that valence ratings were used in place of arousal ratings.

### Results

#### Validity check of valence ratings

A paired-samples *t*-test was conducted on valence ratings that participants made during the experiment, comparing positive sentences paired with neutral faces to neutral sentences paired with neutral faces. As intended, participants rated positive sentences as higher in valence (more positive) (*M* = 6.354, *SE* = 0.199) than neutral sentences (*M* = 5.022, *SE* = 0.156), *t*(31) = 7.917, *p* <.001, *d* = 1.399.

#### Effect of order of sentence presentation

In order to examine the influence of emotional sentence order on recognition memory, a one-way Condition (Positive-Neutral, Neutral–Positive, Neutral–Neutral) repeated-measures ANOVA was conducted on recognition memory (hits). This analysis revealed no significant main effect of Condition, *F*(2, 62) = 1.662, *p* =.198, η_p_^2^ =.051.

To examine whether accounting for anxiety or depression symptomology affected the influence of sentence order on memory, two one-way Condition (Positive–Neutral, Neutral– Positive, Neutral–Neutral) repeated-measures ANCOVAs were conducted on recognition memory (hits), one using anxiety (BAI) scores as a covariate and the other using depression (BDI) scores as a covariate. The first ANCOVA, using BAI scores as a covariate, found neither a significant main effect of Condition, *F*(2,60) = 3.025, *p =.*056, η_p_^2^ =.092, nor a significant interaction between Condition and BAI scores, *F*(2, 60) = 2.603, *p* =.082, η_p_^2^ =.080. The second ANCOVA, using BDI scores as a covariate, revealed no significant main effect of Condition, *F*(2, 60) = 2.087, *p* =.133, η_p_^2^ =.065, as well as no significant interaction between Condition and BDI scores, *F*(2, 60) = 0.818, *p* =.446, η_p_^2^ =.027.

#### Exploratory Bayesian analysis of positive valence and memory

**Fig. 3:**
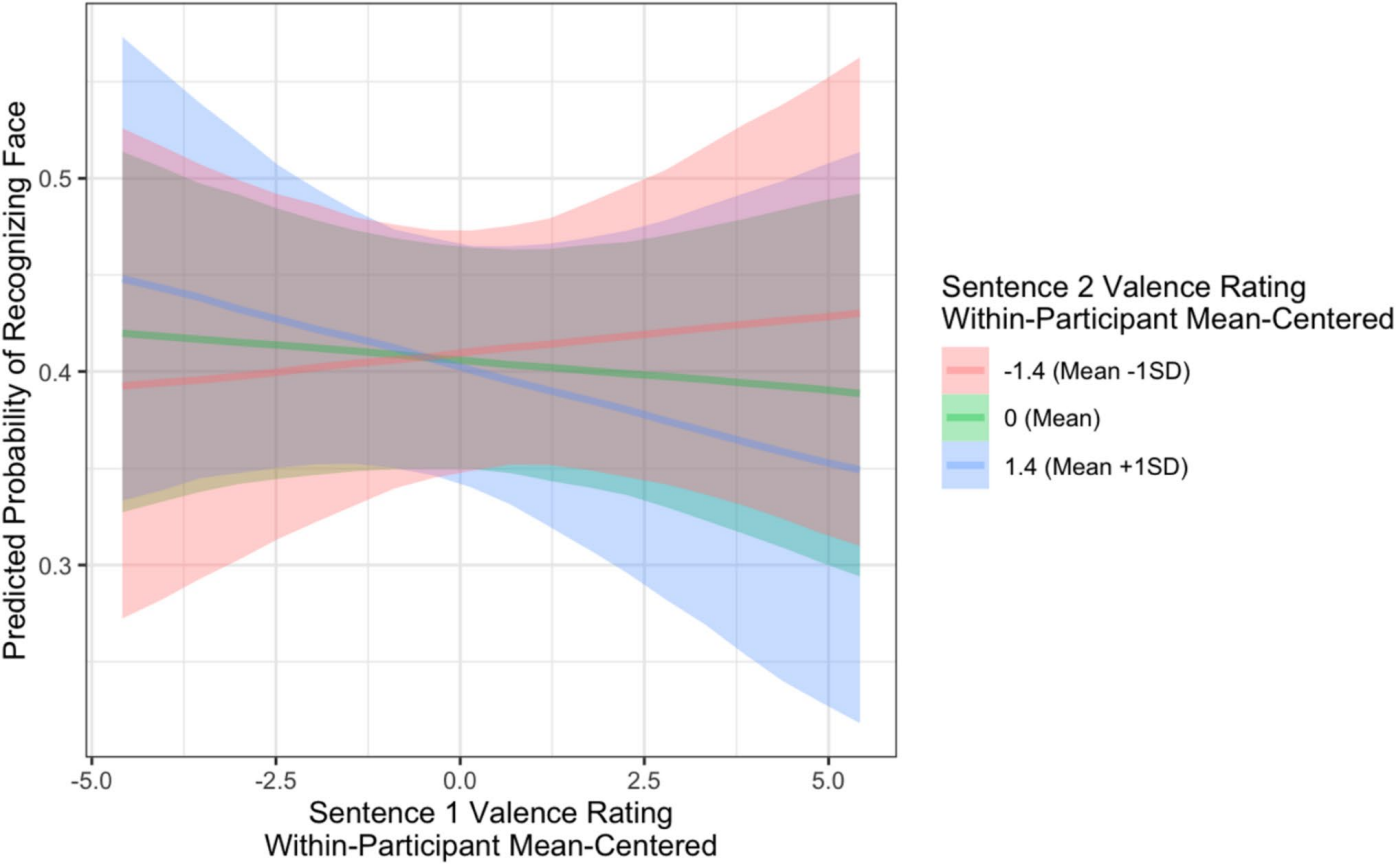
Predicted probability of recognizing a face based on valence ratings for sentences 1 and 2. Predicted probability of a participant recognizing a given face in the positive study, based on their valence ratings for sentences 1 and 2 paired with the face. The y-axis represents the probability of successfully recognizing a face as “old.” The x-axis represents the individuals’ valence rating of the first sentence of a sentence pair. The colored lines represent the degree of positive valence of the second sentence of a sentence pair, with pink indicating low positive valence, green indicating moderate positive valence, and blue indicating high positive valence ratings

Using whether 95% posterior intervals excluded 0 as a rough proxy for significance, we found no significant associations between valence ratings and recognition memory. Valence of the sentence presented at first encoding was not associated with the probability of recognition (β = −0.012, 95% PI [−0.079, 0.053]; OR = 0.99, 95% PI [0.94, 1.03], see Fig. 3), nor was valence of the sentence presented at second encoding associated with the probability of recognition (β = −0.011, 95% PI [−0.075, 0.054], OR = 0.98, 95% PI [0.94, 1.03]). There was no interaction between valence of sentences 1 and 2 (β = −0.020, 95% PI [−0.075, 0.032]).

### Discussion

The findings presented here suggest that the order in which emotional contextual information is encoded is largely irrelevant, regardless of whether that information is negative or positive in valence. This contradicts our hypotheses that recognition memory would be best for faces paired with Negative–Neutral and Neutral–Positive sentences in Experiments 1 and 2, respectively. However, there appear to be several exceptions to this that suggest that further exploration is warranted. In addition, exploratory Bayesian analyses suggested that the arousal rating – but not level of positive valence – of a sentence is associated with reducing the probability of recognizing the face paired with that sentence.

#### Order of negative context sentences did not influence face memory

Contrary to our hypothesis, there was no evidence that the presentation order of negative contextual information influenced memory. However, we observed an interaction between condition and sex of the participant such that, for females, recognition memory was better for faces in the Negative–Neutral and Neutral–Negative conditions as opposed to the Neutral–Neutral condition (see ESM). This indicates that the negative sentences used here were emotionally arousing enough to have an impact on memory, at least for females. In addition, this finding suggests that for females, the presence of negative information, but not the order in which it was presented, enhanced memory.

It must be mentioned that these results differ from those predicted by the emotional carry-over effect, namely that blocks of neutral stimuli are more likely to be remembered when following a block of negative stimuli than when following a block of neutral stimuli (Tambini, Rimmele, et al., 2017). However, as previously mentioned, the procedural timing used in the present study differed from that used in Tambini, Rimmele, and colleagues (2017). In the latter, blocks lasted for approximately 22 min and were separated by valence (i.e., negative and neutral stimuli were not mixed). Such a design may have allowed for participants to reach a state of higher physiological arousal than the brief and mixed presentation of stimuli used by the current study could induce. Further, the lack of observed “carry over” of arousal may have been due to increased stimuli distinctiveness caused by the current study intermixing negative and neutral sentences. This intermixing of stimuli may have enhanced the distinctiveness of negative stimuli by allowing for an immediate and direct comparison to neutral stimuli (Talmi et al., 2007).

#### Faces paired with higher arousal sentences were less likely to be remembered than lower arousal sentences

Exploratory Bayesian analyses suggested that sentence arousal rating at both the first and the second encoding phase was negatively correlated with later face recognition memory, such that faces paired with the most arousing sentences were the least likely to be recognized. It is possible that the presence of a high-arousal sentence biased processing and consolidation towards the sentence and away from the face. This idea of a trade-off between encoding the sentence or the face in a face-sentence pair is supported in part by the results of a follow-up study (presented in full in the ESM) that found a positive relationship between sentence arousal and sentence recognition (see ESM Fig. 2). This interpretation is further supported by the *arousal-biased competition and the GANE model*, which contends that arousal can modulate the strength of competing representations, thereby biasing attention and consolidation via norepinephrine hotspots of the most high-priority, goal-relevant pieces of information (Mather & Sutherland, 2011; Mather et al., 2016). Though participants were told to remember both the face and the sentence, they were completing arousal ratings at encoding, which were likely made mostly based on the sentences that varied in arousal, instead of the neutral faces. Thus, the faces may have been lower priority and less goal relevant, and when a sentence evoked higher arousal, this may have led to lateral inhibition and less cognitive processing of the memory representations for the face. Indeed, studies have shown that consolidation can also play a role in memory binding such that arousal only enhances the consolidation of contextual or temporal information if it is initially integrated into the event model (Bennion et al., 2015; McClay et al., 2023; Takashima et al., 2007). This would make it likely that for the high-arousal sentences, participants may have failed to bind their memory of the sentence to their memory of the face, meaning that the increased arousal elicited by negative sentences did not transfer to the encoding of neutral sentences. If highly arousing negative sentences were less likely to be bound to the paired face, then order would be less likely to influence memory.

This is also consistent with findings that associative memory and contextual binding are weakened for negative and arousing information (e.g. Bisby et al., 2018; Bisby & Burgess, 2014; Madan et al., 2012, 2017). Moreover, this suggested impairment in associative encoding for negative arousing information has been associated with increased coupling between the ventral-lateral amygdala and the central amygdala and lack of extra-hippocampal support at encoding (Madan et al., 2017). In practice, when encountering a sentence that evoked high arousal, participants may have focused on the sentence alone without linking it to the face, such that the face alone at retrieval did not act as a sufficient cue for either recognition or recall memory.

Furthermore, the lack of an interaction between the arousal ratings of sentences 1 and 2 further suggests that the order in which negative contextual information was presented was irrelevant, with the mere presence of highly arousing negative information being enough to impair recognition memory.

The lack of a relationship between level of positive valence and recognition memory may be explained by the broadened scope of attention commonly associated with positive moods or stimuli (see Conway et al., 2013) preventing the impairment of binding seen with arousing negative information. Additionally, it has been suggested that positive words enhance associative memory only when both words are perceived as positive at encoding (Madan et al., 2019; Zimmerman & Kelley, 2010). Future studies could explore if this is also the case in a face-sentence paradigm.

While our exploratory Bayesian analyses found that higher arousal ratings of a sentence were associated with lower recognition memory of the face presented with that sentence, an ANCOVA found that female participants demonstrated decreased recognition memory for faces in the Neutral–Neutral condition, as compared to the conditions that included emotional sentences (i.e., E–N and N–E), while males showed no differences in recognition memory based on condition. The analyses differed in their approach to both sentence ratings and sentence pairings, with the ANCOVAs using our normative ratings and considering both sentence-face presentations as a pair, and the Bayesian analyses using each participant’s rating of each individual sentence as a separate instance. Thus, the increased recognition of faces paired with negative sentences by female participants may not be specifically driven by higher arousal. Individual differences in the experience of emotion likely influenced each individual’s memory (see Hamann & Canli, 2004; Montagne et al., 2005).

## Limitations

The finding that the magnitude of positive valence was not associated with any differences in recognition memory without controlling for anxiety may be related to stimulus selection. Primarily, it has previously been shown that lower variance in discriminability of positive stimuli, as compared to discriminability of negative stimuli (Inaba et al., 2005), makes it difficult to match stimuli on arousal while retaining external validity. For this reason, and to avoid the use of taboo sentences for the positive study, which can introduce variance in participants’ interpretation, arousal levels were not matched across experiments. As such, the combination of increased arousal levels in the negative study and the finding that arousal predicted recognition memory may indicate that arousal levels are a better predictor of recognition memory than level of positive valence. However, it is likely a more complicated story, as studies have shown that the effect of arousal differs depending on valence. For example, arousal increased amygdala connectivity to the inferior frontal gyrus and middle occipital gyrus for negative stimuli, but decreased it for positive stimuli (Mickley Steinmetz et al., 2012). Future studies could examine this question by systematically varying valence and arousal levels of the stimuli in a similar paradigm.

The current study included both social and non-social descriptors and actions for the person-based contextual information. Future studies could deliberately vary social and non-social sentences in order to examine whether social relevance accounts for some of the variance in memory performance. In addition, some studies have indicated that participants may quickly form trait impressions, such as trustworthiness or dominance, when viewing unfamiliar faces, even when the faces have neutral expressions (Sutherland & Young, 2022; Todorov & Oh 2021). It follows that these spontaneous impressions may influence one’s later memory of the person-based information. Thus, although the current study randomized sentences to avoid various context-based impressions of faces, future studies could deliberately investigate the influence of spontaneous impressions on memory for faces and person-based information.

Relatedly, our use of sentence-face pair randomization meant that we could not control for the valence and/or arousal of sentences immediately preceding or succeeding other sentences, opening the door to possible cross-trial contamination. In the current study, analyses of the emotional value of the sentences that came before or after each face indicated no impact on the memorability of each face. However, future studies could consider forgoing or modifying the randomization methodology in favor of a more controlled encoding order. In addition, some studies have used varying old images at test (such as the same faces at a different angle) to make the test more difficult. Though Mattarozzi and colleagues (2019) did not find differences in memory when faces were shown at a different angle, a design such as this would allow for distinctions to be made based on memory for the identity of the face as opposed to the specific image. Future studies could add in this variable as part of the study design.

Lastly, participants were at floor in their ability to recall the sentences when cued by the face 1 week after encoding. This is likely due to the longer consolidation period in this study as compared to most other recall memory studies. Such a conclusion is supported by Mattarozzi and colleagues (2019), who found that emotional sentences only influenced recognition memory after a 1-week delay, as compared to an immediate-recognition and immediate-recall test; recall memory was not tested after a 1-week delay, so no comparison is available. In addition, Knight and Mather (2009) found retrograde enhancements in memory coming before emotional oddball stimuli after a 1-week delay, but not in tests that occurred immediately after encoding. Longer consolidation periods may enhance emotion’s influence on memory, since sensory details and implicit memory for salient emotional stimuli may be less likely to be disrupted over time (Bowen et al., 2018; Knight & Mather, 2009). Due to this effect, we may have observed the implicit effects on recognition memory despite participants being unable to explicitly recall the sentence. Future studies could consider examining comparing differences in both recall and recognition immediately as compared to at a longer delay.

## Conclusions

We found no evidence that the presentation order of negative contextual information influenced facial recognition memory, with the exception that females displayed increased recognition memory when a negative sentence was present, regardless of whether it preceded or followed a neutral sentence. Likewise, there is insufficient evidence to suggest that the order in which positive sentences were presented affected recognition memory. Nonetheless, exploratory Bayesian analyses showed that the arousal level of a sentence was directly associated with a reduced probability of later recognition of the face paired with that sentence, such that higher arousal led to worse recognition memory. Therefore, we believe that arousal influenced memory in the Negative experiment by decreasing memory for the associated face, likely by biasing attention towards the highly arousing sentence and away from the less arousing face, or by disrupting the binding between the face and the sentence at encoding.

## Electronic supplementary material

Below is the link to the electronic supplementary material.

Supplementary file1 (DOCX 279 KB)

## Appendix

Sentences listed by valence in female form. Male versions of each sentence were also used for different participants as part of the counterbalancing scheme.

## Female Negative

She hit him with her car.

She is an alcoholic.

She blew up the building.

She is possessed by a demon.

She mutilated the body.

She wished he had cancer.

She raped the boy.

She is a terrorist.

She slaughtered the baby.

She gave someone syphilis.

She abuses her child.

She suffocated her wife.

She killed the child.

She owns slaves.

She hurt her neighbor.

She insulted a young boy.

She stabbed a bunny to death.

She assaulted a student.

She hates puppies.

She poisoned her spouse.

She is a danger to society.

She crushed the girl's skull.

She shot people in church.

She drowned a kitten.

She blames the victim.

She spit in her face.

She worships the devil.

She has been to prison.

She upset the child.

She caused a car accident.

She despises her son.

She is a pervert.

She wounded a baby.

She destroyed the house.

She is a traitor to her nation.

She held hostages at gunpoint.

She humiliated a little boy.

She crashed her friend's car.

She doesn't move for ambulances.

She gets angry when drunk.

She pointed a gun at someone.

She lies to her spouse.

She hired a hooker.

She burned down a building.

She called her daughter ugly.

She damaged the new furniture.

She has evil thoughts.

She wishes he was dead.

She assassinated the president.

She gets mad at babies.

She slapped her face.

She is cruel to animals.

She is guilty of kidnapping.

She made her get an abortion.

She is a controlling girlfriend.

She screamed at a child.

Her family describes her as evil.

She makes people fearful.

She is defiant to her boss.

She invokes chaos at home.

## Female Neutral in Negative Study

Her brother is in the army.

Her car has a trunk.

Her fireplace has a mantel.

Her hair used to be blond.

Her outfit was plain.

Her pants are blue.

Her sister is a nurse.

She ate a salad.

She ate an egg for breakfast.

She ate with a fork.

She attends the event.

She bought a memory card.

She bought an antique clock.

She bought curtains for the house.

She buttered her bread.

She buys a kitchen appliance.

She buys a plane ticket.

She camps outdoors in tent.

She collects rocks for a project.

She cooks on a stove.

She cooks the mushrooms.

She cuts the grass.

She drew a square on paper.

She drinks milk.

She drives a car to school.

She drove down the highway.

She eats dinner with friends.

She eats pizza while watching TV.

She eats soup from a bowl.

She eats the pie.

She excavated the statues.

She explored the cottage.

She fries fish for the cookout.

She gets the mail.

She has a key.

She has many opinions.

She has one dollar.

She held the crown.

She is a piano teacher.

She is a quiet woman.

She is a writer.

She is curious about geology.

She is fascinated by spoons.

She learns about a new method.

She left her seat to go home.

She likes the color yellow.

She likes to eat grape jelly.

She listened to a theory.

She listens to the orchestra.

She lives on Queen Avenue.

She looked at a tree.

She looks at the sky.

She mowed the lawn.

She named the restaurant.

She opened the door.

She opened the locker.

She opens the bottle.

She opens the window.

She operated the machine.

She owns a green shirt.

She owns a house plant.

She owns a sailboat.

She owns a trumpet.

She painted the walls.

She photographs the mountain.

She plays the violin.

She pointed with her finger.

She printed the news.

She pulls the wagon.

She purchased fabric to make clothing.

She ran an errand.

She read a passage.

She reads the contents.

She relaxes in her home.

She rode the bus.

She sailed the ship.

She sat in the chair.

She sat on the bench.

She saw a hawk.

She saw an inactive volcano.

She sees a frog.

She shakes her boss' hand.

She shaves with a razor.

She sleeps in the hotel.

She stacks hay.

She tied a knot.

She took a taxi into town.

She turned on the lamp.

She used a lantern.

She used an umbrella.

She used the bathroom earlier.

She visits the museum.

She walked down a corridor.

She was serious about the test.

She watched a beehive.

She watches the clouds.

She waved the flag.

She wears a tank top.

She went to a nude beach.

She went to the circus.

She went to the prairie.

She wore a hat.

She works in a skyscraper.

She works on the computer.

She wrote her friend a letter.

She wrote in the journal.

She adopted a kitten.

She collects coins.

She cracked an egg.

She discovered a star.

She drinks wine.

She eats dinner with family.

She is humble when praised.

She is indifferent.

She lives on the coast.

She produced a medicine.

She reads poetry.

She runs errands with her husband.

She stayed in a hotel.

She watched tennis.

## Female Neutral in Positive Study

Her brother is in the army.

Her car has a trunk.

Her fireplace has a mantel.

Her hair used to be blond.

Her outfit was plain.

Her pants are blue.

Her sister is a nurse.

She ate a salad.

She ate an egg for breakfast.

She ate with a fork.

She attends the event.

She bought a memory card.

She bought an antique clock.

She bought curtains for the house.

She buttered her bread.

She buys a kitchen appliance.

She buys a plane ticket.

She camps outdoors in tent.

She collects rocks for a project.

She cooks on a stove.

She cooks the mushrooms.

She cuts the grass.

She drew a square on paper.

She drinks milk.

She drives a car to school.

She drove down the highway.

She eats dinner with friends.

She eats pizza while watching TV.

She eats soup from a bowl.

She eats the pie.

She excavated the statues.

She explored the cottage.

She fries fish for the cookout.

She gets the mail.

She has a key.

She has many opinions.

She has one dollar.

She held the crown.

She is a piano teacher.

She is a quiet woman.

She is a writer.

She is curious about geology.

She is fascinated by spoons.

She learns about a new method.

She left her seat to go home.

She likes the color yellow.

She likes to eat grape jelly.

She listened to a theory.

She listens to the orchestra.

She lives on Queen Avenue.

She looked at a tree.

She looks at the sky.

She mowed the lawn.

She named the restaurant.

She opened the door.

She opened the locker.

She opens the bottle.

She opens the window.

She operated the machine.

She owns a green shirt.

She owns a house plant.

She owns a sailboat.

She owns a trumpet.

She painted the walls.

She photographs the mountain.

She plays the violin.

She pointed with her finger.

She printed the news.

She pulls the wagon.

She purchased fabric to make clothing.

She ran an errand.

She read a passage.

She reads the contents.

She relaxes in her home.

She rode the bus.

She sailed the ship.

She sat in the chair.

She sat on the bench.

She saw a hawk.

She saw an inactive volcano.

She sees a frog.

She shakes her boss' hand.

She shaves with a razor.

She sleeps in the hotel.

She stacks hay.

She tied a knot.

She took a taxi into town.

She turned on the lamp.

She used a lantern.

She used an umbrella.

She used the bathroom earlier.

She visits the museum.

She walked down a corridor.

She was serious about the test.

She watched a beehive.

She watches the clouds.

She waved the flag.

She wears a tank top.

She went to a nude beach.

She went to the circus.

She went to the prairie.

She wore a hat.

She works in a skyscraper.

She works on the computer.

She wrote her friend a letter.

She wrote in the journal.

She buys ketchup for hamburgers.

She crossed the street.

She filled a jug with water.

She filled the icebox.

She met a nun.

She owns a field to farm.

She passed a fire hydrant.

She sees a lighthouse.

She taught history at her school.

She updates the score board.

She was born in the fall.

She wore a vest.

She works at the office.

She wrote a paper.

## Female Positive

She is a patriot for her country.

She advocates for civil liberties.

She is an honest person.

She received a promotion.

She feels loved by her family.

She earned a diploma.

She got an endorphin rush from exercising.

Her jokes made everyone laugh.

She bought a diamond ring.

Her family gives her much joy.

She is a happy person.

She is a good mother.

She has many great successes.

She triumphed in the Olympics.

She discovered a treasure.

She married her high school sweetheart.

She won big at the casino.

She felt truly alive.

She cheers for her children.

She got a huge cash bonus.

She is thankful.

She bought her mother a house.

She is optimistic about life.

She is a terrific parent.

She performed a miracle.

She's a devoted wife.

She called the girl beautiful.

She wishes people a Merry Christmas.

She is friendly.

She is a champion.

She beamed with pride.

She got engaged.

She is an outstanding person.

She was an incredibly confident woman.

She is a millionaire.

She protected her son.

She was accepted into college.

She is a thoughtful person.

She was gentle with the baby.

She's a joy to be around.

She is respectful.

She got a new pet for Christmas.

She has ambition.

She won a trophy.

Her personality is radiant.

She won the lottery.

She just got a new puppy.

She adopted a baby.

She is debt free.

She bravely rescued a cat from the fire.

Her first kiss was exciting.

She is kind to everyone.

She is loyal to her friends.

She has a good sense of humor.

She owns a yacht.

She cooked her husband dinner.

She gives good hugs.

She performed a great act of rescue.

She inspired others with her resilience.

## Declarations

### Author Note

The preregistration for this study can be found here: https://osf.io/v5z93

Materials, data, and analysis code can be found here: https://osf.io/rhac3/

Katherine R. Mickley Steinmetz: https://orcid.org/0000-0001-9576-9733
