# Supplementary material for: Investigating memory for faces based on emotional contextual information
Source: Mem Cognit. 2026 Mar 30;54(5):1650–66. doi: 10.3758/s13421-025-01830-w (PMC13407620; doi:10.3758/s13421-025-01830-w)
Supplement: Supplementary file 1 — Supplementary file1 (DOCX 279 KB) [file 13421_2025_1830_MOESM1_ESM.docx]

**Supplementary Methods**

**Power Analysis**

We conducted iterative simulation-based power analyses using R statistical software (V4.2.1; R Core Team, 2021). Each individual simulation assumed 90 faces encoded and 90 new lure faces at retrieval, a mean hit probability (for neutral-neutral encoded faces) of 0.6, and a mean false alarm probability of 0.2. Effects of encoding condition were simulated such that hit rate was highest on average in the negative-neutral condition followed by the neutral-negative condition, and lowest in the neutral-neutral condition. Effects of encoding condition on hit rate were allowed to vary across participants according to a uniform distribution (see attached code for details), and effects of the negative-neutral encoding order were assumed independent of effects of the neutral-negative encoding order across participants. Single-trial data were simulated using a Bernoulli distribution (e.g. 1 = “choose old”, 0 = “choose new”), then aggregated to participant-level summaries for each condition to run the ANOVA analysis reported in the main text methods section. Simulations were conducted for η_p_^2^ values ranging from 0 to 0.3, with 2000 iterations per effect size. For each effect size, the proportion of iterations with *p* < .05 for the effect of condition was taken as the approximate power.

**Supplementary Data and Analyses**

**Negative Study: Sex Differences in Recognition Memory Based on Order**

To determine if sex of picture had an effect on recognition memory, a mixed factorial Sex of Picture (Male, Female) x Sex of Participant (Male, Female) x Emotion (Negative-Neutral, Neutral-Negative, Neutral-Neutral) ANOVA was performed on picture memory. There were no main effects. There was an Emotion x Sex of Participant interaction, *F*(1,30) = 3.469, *p* = .038. Post hoc *t*-tests revealed that female participants remembered faces with negative-neutral sentence order (*M* = 0.577, *SE* = 0.043) more than neutral-neutral sentence order, (*M* = 0.498, *SE* = 0.043), *t*(15) = 2.195, *p* = .004. Female participants also remembered faces with neutral-negative sentence order (*M* = 0.579, *SE* = 0.053) more than neutral-neutral sentence order, *t*(15) = .866, *p* < .05. Female participants showed no significant difference between memory for faces of negative-neutral and neutral-negative sentence order. Male participants showed no difference for facial memory between different sentence order (see Supplemental Figure 1).

**Supplemental Figure 1**

*Sex Differences in Recognition Memory Based on Order*


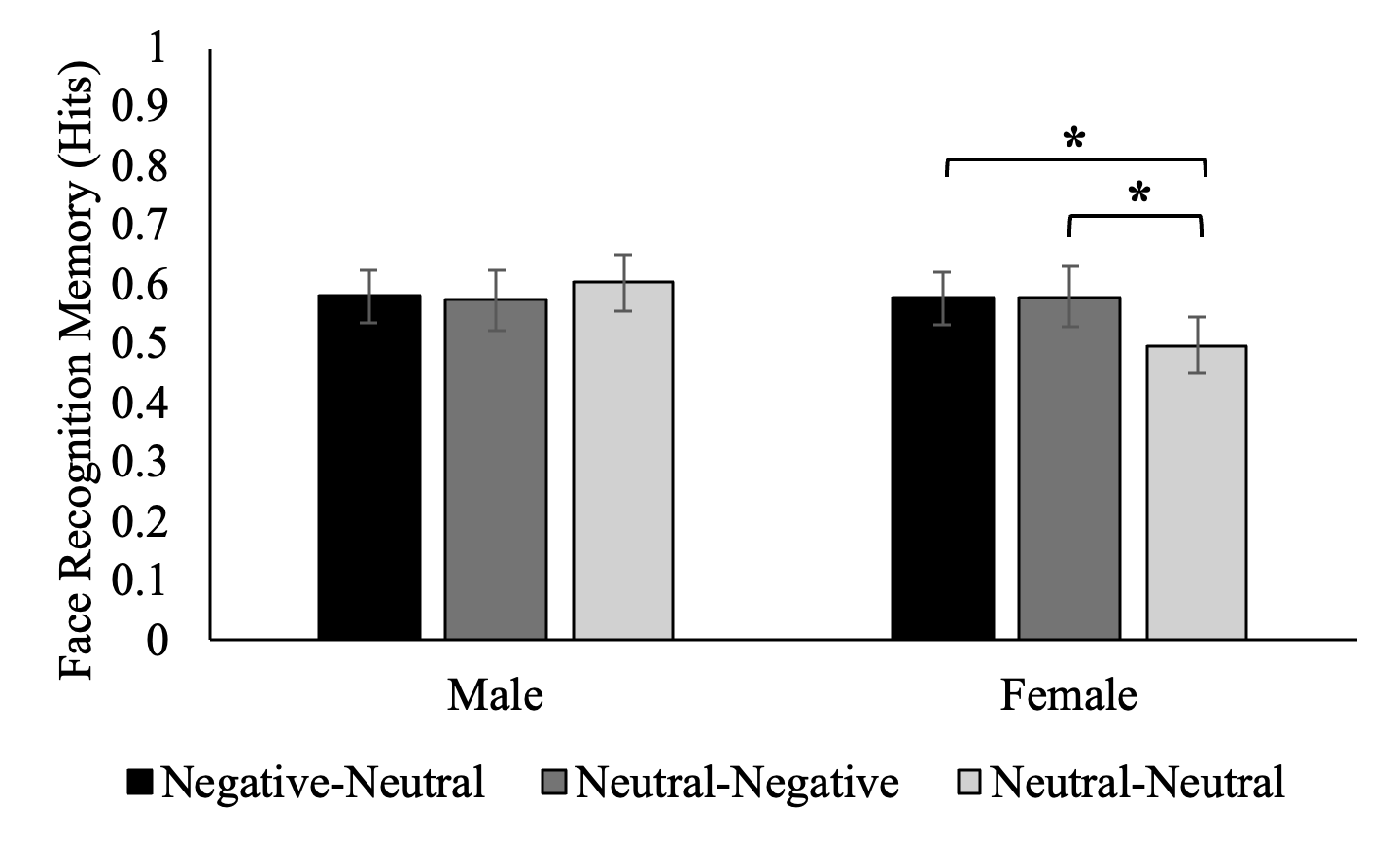


*Note.* Recognition memory (hits) for faces paired with either Negative-Neutral, Neutral-Negative, or Neutral-Neutral sentence pairs, separated by sex. Post-hoc *t*-tests indicated that female participants showed enhanced recognition memory for faces paired with negative information (Negative-Neutral and Neutral-Negative) as compared to only neutral information (Neutral-Neutral). This effect was not seen in males. The asterisk (*) indicates *p* < .05. Error bars represent standard error.

**Negative Study: Within-Participant Correlations Between Arousal Ratings and Recall Probability**

To supplement primary multilevel model analyses for arousal ratings with more interpretable metrics, we computed correlations for each participant individually between arousal ratings (separately for ratings for the sentences presented at first versus second encoding) and recognition probability. Then, one-sample *t*-tests were used to understand whether such correlations differed from 0 on average. Confirming the direction of primary results, mean correlation between arousal and recognition probability was negative for both the sentence at first encoding (*p* = -.13) and second encoding (*p* = -.06). However, this mean correlation was not significantly different from 0 for either the first (*t*(30) = -1.91, *p* = .065, Cohen’s d = -0.34) or second sentence (*t*(30) = -0.82, *p* = .419, Cohen’s d = -0.15). We note that we focus on the multilevel model in the main text because it appropriately accounts for the nested structure of the data (trials within participants). In contrast, an overall or individual-level correlation conflates within- and between-participant variance and fails to account for differences in trial counts and arousal score distributions (Bloom et al., 2022). For example, participants with only one trial at a given arousal level exert disproportionate influence in correlation analyses, whereas multilevel models handle such imbalances through appropriate weighting.

**Follow Up Study to Assess Reaction Times**

In order to determine if participants spent time processing the face, or if they only focused on the sentences, we conducted a comparison reaction time study where just the sentences were presented. This could then be compared to the reaction times in the negative study. If the reaction times for making the arousal rating were significantly longer in the negative study as compared to when sentences were presented alone, this would indicate that some processing time was likely spent attending to the face before making the arousal rating.

***Participants***

Twenty-two female participants aged 19-21 years old (*M* = 20.636, *SE* = 0.581) participated in this study. For all participants English was their first language.

***Materials***

The same sentences that were used in the Negative Study were presented in a randomized order.

***Procedure***

Each sentence was presented on screen for 8 seconds. Participants rated the arousal of each sentence using the same scale as in the Negative Study.

***Results***

An independent samples *t*-test indicated that participants in the Negative Study (who viewed faces with sentences) took significantly longer to complete arousal ratings (*M* = 3.997, *SE* = 0.103) than participants that viewed the same sentences alone (*M* = 3.054, *SE* = 0.135), *t*(52) = 5.613, *p* < .001.

**Follow Up Study to Assess Memory for Sentences**

To assess differences in sentence memorability according solely to their arousal level, we conducted a comparison study in which participants viewed the sentences alone at encoding (i.e., without a paired face) and then completed a recognition memory test for the sentences one week later. A positive relationship between sentence arousal and sentence recognition would support the idea of a trade-off between encoding the sentence or the face in a face-sentence pair, as was presented to participants in the main study discussed in this article.

***Participants***

Twenty-two participants (16 female) aged 20-33 (*M* = 23.091, *SE* = 0.680) completed this study. For all participants, English was their first language.

***Materials***

This follow-up study used the exact same sentences as presented in the Negative Study, such that 60 negative valence sentences and 120 neutral valence sentences were utilized. Participants viewed half of the total sentences (i.e., 30 negative and 60 neutral) presented in a random order at encoding, counterbalanced across two groups. All 180 sentences were presented in a randomized order to each participant during the recognition memory test.

***Procedure***

During the encoding phase, each sentence was presented on screen for 8 seconds. Participants rated the arousal of each sentence using the same scale as in the Negative Study. One week later, participants were shown the sentences that they saw at encoding mixed with new sentences and were asked to indicate if the sentence was old or new.

***Data Analysis***

Multilevel logistic regression models were used to estimate associations between sentence arousal at encoding and probability of later recognition via the recognition test. Models included random intercepts and slopes for the arousal term for each participant. New sentences not presented at encoding were not included in these models. Arousal at encoding was within-participant mean centered to isolate within-person effects of arousal without confounding based on between-participant differences in average arousal. Recognition was coded as 1 (successful recognition) if a participant rated as sentence as previously seen, and 0 (unsuccessful recognition) if a participant rated a sentence as new during the recognition test.

***Results***

Multilevel logistic regression indicated that higher sentence arousal at encoding was associated within-participants with a higher likelihood of recognition, such that a 1SD increase in arousal (above mean) was associated with 1.81-fold greater odds of recognition (95% CI [1.48, 2.22]; Supplemental Figure 2).

**Supplemental Figure 2**

*Probability of Correct Sentence Recognition as a Function of Arousal at Encoding*
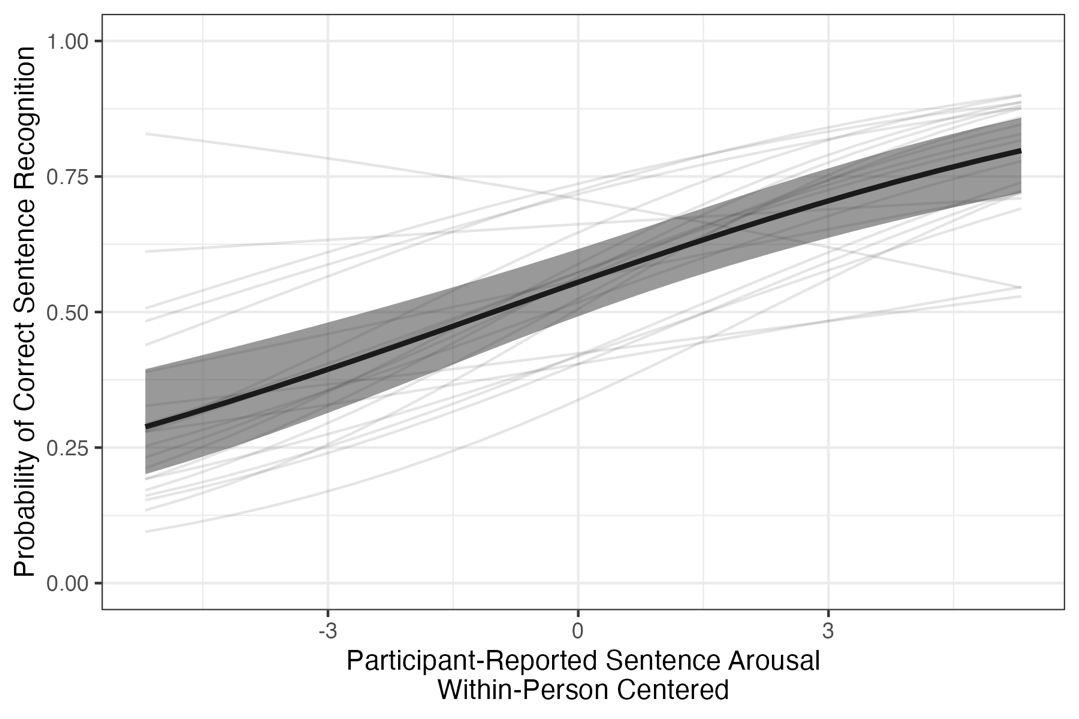


*Note.* The x-axis shows within-participant centered arousal at encoding (0 represents a participant mean arousal across all sentences), and the y-axis indicates model-predicted probability of correct recognition. The thick line shows estimated group-level logistic multilevel regression predicted probability of recognition, with the shaded area indicating a 95% confidence interview. Thin lines show participant-specific marginal model predictions.

**Both Studies: Differences in Recognition Memory Based on Anxiety**

A mixed measures ANCOVA was conducted on correctly recognized faces (hits) across Condition (Emotional – Neutral, Neutral – Emotional, Neutral – Neutral) x Study (Negative Experiment, Positive Experiment) with the Beck Anxiety Inventory (BAI) as a covariate. A significant interaction between BAI score and condition was found, *F*(2, 122) = 3.656, *p* = .029, η_p_^2^ = .057. Post-hoc correlations revealed that higher anxiety scores were correlated with higher corrected recognition memory hits for Emotional – Neutral order, *r*(63) = .260, *p* = .038. Anxiety scores were not correlated with recognition hits for Neutral – Emotional (*r*(63) = .048, *p* = .706) or Neutral - Neutral (*r*(63) = .050, *p* = .693) contextual order.

**References**

Bloom, P.A., Thieu, M.K.N., Bolger, N. (2022). Commentary on Unnecessary reliance on multilevel modelling to analyze nested data in neuroscience: When a traditional summary-statistics approach suffices. *Curr Res Neurobiol, 3,* 100041. doi: 10.1016/j.crneur.2022.100041. PMID: 36685767; PMCID: PMC9846465.

R Core Team (2021). R: A language and environment for statistical computing. R Foundation for Statistical Computing, Vienna, Austria. https://www.R-project.org/.
